# Editorial: severity in a genomic age

**DOI:** 10.1038/s41431-024-01766-w

**Published:** 2025-02-19

**Authors:** Felicity Boardman

**Affiliations:** Applied Health Directorate, Warwick Medical School, Gibbet Hill Road, Coventry, CV4 7AL UK

**Keywords:** Health policy, Medical ethics

The ostensibly simple, yet overwhelming complex, notion of condition severity is employed across genomic and reproductive medicine in various ways. Most significantly, as a gatekeeper for access to a range of reproductive and genomic technologies (e.g. prenatal testing and abortion, PGD, carrier/newborn screening). Whilst lacking definition, severity is frequently employed to interpret the (anticipated) impacts of genetic conditions, and consequently contributes to decisions on which variants are included on screening panels [1, 2] and/or returned as incidental findings [3]. With the incremental ‘mainstreaming’ [4] of genomics into the domain of population health across the globe, the way that condition severity is interpreted and harnessed within screening programme design renders it relevant to the lives of increasing numbers of people.

This parallel burgeoning recourse to severity, alongside its lack of conceptual structure provided the impetus for an international workshop, hosted by the Brocher Foundation, Switzerland, in June 2023 [5]. Led by a small group of scholars and researchers independently working on the concept in three different countries (UK, Canada and Australia), the workshop was designed as a series of deliberative discussions and knowledge consolidation activities focusing on ‘severity’ across its various applications and contexts (e.g. healthcare policy, clinical practice, population health, technologies) and through different interpretative lenses (e.g. from the perspectives of clinicians or those with lived experience). The workshop was attended by an international group of 22 researchers, scholars, clinicians, members of advisory/ policy/advocacy groups and charities from ten different countries, and the discussions that emerged from it provided a launch platform for this special issue.

The aim of this collection is to showcase the breadth and depth of research and scholarship on the concept of ‘severity’ as it is mobilised within the field of genomic medicine, and to highlight the various nexus points between healthcare policy, law, clinical practice and social life that it directly shapes.

In the first instance, this collection offers an important clarification of terminology [6, 7]. While ‘severity’ and ‘seriousness’ are frequently used interchangeably in the literature, by distinguishing between the two, the lenses through which they are constructed are rendered visible [8]. ‘Severity’, as noted by Dive et al. [7], most commonly appears as medical terminology to describe the ‘clinical features’ (7: 2) of a genetic condition, as part of a graded spectrum: mild/moderate/severe. ‘Seriousness’, on the other hand, is a colloquial word, less anchored to medical terminology, that denotes the *impact* a condition has on a person’s life. This includes their health and functional status, but also their psychosocial well-being, and the social, cultural, political and environmental context that frames their lives.

Whilst the concepts can be mutually constituting to varying degrees, by distinguishing between them, it is possible to conceive of conditions that are simultaneously ‘mild’ but nevertheless ‘serious’ and vice versa. This dis-entangling of severity from seriousness sets the stage for this special issue, opening up a critical space for their re-examination.

Kleidermann et al.’s paper [6], emerging from both previous work in this area [9, 10], and deliberations catalysed through the Brocher workshop, levers off the severity and seriousness distinction, offering conceptual scaffolding to uphold a transferable framework through which seriousness can be accessed from a range of standpoints. By not defining seriousness, but instead outlining its constituent parts (which, notably, include clinical severity), and the symbiotic relationships between them, the framework is designed to provide a roadmap for discussions around seriousness across the full range of contexts it appears.

In the wider context of poor understanding of penetrance for many genetic variants (particularly at population level) [10], the challenges of deriving prognostic information from genetic findings alone (see Vona’s paper [11] on ‘mimic genes’ in this issue) along with the wide spectrums of presentation and lived experience associated with genetic conditions (as described by Kriukelis et al. [12], Vona [11] and Freeman et al. [13] within this collection), Kleidermann et al. [6] underscore the need for wide parameters when considering which factors are relevant to an appraisal of seriousness.

This panoramic view of seriousness is particularly important, as Swainson et al. [14] highlight, because social, psychological and cultural conditions play a large part in its construction. The very inclusion of a condition on a screening panel, for example, was found to be interpreted as an a priori judgement on its severity by parents in their study, all of whom had undergone carrier screening and received a result indicating a high chance of having a child with a genetic condition (Swainson et al, 2024). Notably, when faced with a highly variable genetic condition, the high chance couples also focused on the ‘worst-case’ (i.e. the most clinically severe presentation) to inform how they responded to the result. The possibility of milder phenotypes were largely excluded from their deliberations precisely because of the ambiguity they introduce, and the impact this, in turn, would have on their ability to make decisions [14].

Although there may be greater tolerance of uncertainty with regards to severity and seriousness in other decision-making contexts (e.g. healthcare policy), the possibility of severity and seriousness being syphoned down to their extremes (severe/mild) as a device to simplify highly complex reproductive decisions with lots of ‘grey areas’, raises important questions about whether and how high quality and accurate information about conditions can be imparted to (prospective) parents facing these decisions, and how the question of seriousness could be broached in a way that facilitates rather than complicates reproductive decisions.

The testimonies of people living with genetic conditions have long been seen as offering a bridge between severity and seriousness, and there have been moves to amplify these voices within deliberations around severity [9, 13]. Kaur’s [15] paper, for example, reinforces the need for multi-dimensionality in order to access seriousness. More specifically, the paper demonstrates the links between the seriousness of a condition, and the degree to which it impacts a person’s mental health and quality of life. Whilst quality of life is similarly a difficult concept to capture (given that it is highly contingent and context-sensitive), its intersections with related concepts, such as severity and seriousness, do warrant further exploration, especially when they are used as thresholds for technology access. Gallois et al. [16] in this issue, for example, review 216 articles where ethical criteria for prenatal testing access are outlined. They note that while severity itself is never defined (despite it being the indication for technology use), six core recurring characteristics of a condition can be used as ‘proxies’ for severity, characteristics which can be transferred across prenatal contexts. Some of these proxies relate to the experience a person has with a genetic condition (e.g. age of onset, quality of life), the properties of the genetics involved (penetrance, monogenetic aetiology), but also the clinical context (absence of treatment), demonstrating once again the complex range of factors involved in determining condition severity and seriousness.

Whilst categorisation systems can be built around such proxies of severity [17], Taylor-Sands et al. [18] nevertheless argue that such categorisations should not, in themselves, be used to limit access to technologies such as NIPT. Indeed, labelling a condition as ‘severe’ or ‘serious’ can not only stigmatise the condition and, by extension, people with the condition, it can also reduce the reproductive autonomy of prospective parents. Taylor-Sands et al. suggest an alternative approach to filtering access to technologies such as NIPT- a focus on information provision, consent and counselling to ensure that decisions to use a technology are made in line with a person’s values and context. Co-produced information tools, as an adjunct to genetic counselling can support such decision-making (for example, see King et al. [19],) and enable pregnant people to make decisions about how much information they want or need, without the need to label conditions.

While Taylor-Sands et al. highlight the limitations on reproductive autonomy when severity is used to unlock access to NIPT, Nov-Klaiman et al. [20], in their study of prenatal testing in Germany, explore the impacts when it is seemingly absent. In Germany, Nov-Klaiman et al. note, condition severity is conspicuously missing from policy and legislation that governs access to prenatal testing and pregnancy termination, despite its explicit *presence* as a criterion governing access to PGD [21]. Whilst severity considerations invariably filter into decisions, it is ultimately health professionals in this context who sanction access to them, grounding their decisions in judgements on the imagined impacts on the woman’s physical and psychological health should a pregnancy (with a foetus diagnosed with a condition) continue.

Whilst this side-stepping of the severity question (which can be especially uncertain in prenatal contexts- [22, 23]) as a formal part of pregnancy management decisions diminishes the sceptre of eugenics that has long besmirched prenatal testing [20], like Taylor-Sands et al., they note it can also reduce reproductive autonomy. For both Taylor-Sands et al. and Nov-Klaiman et al. therefore, recourse to severity, either in its presence or its absence, confers power to health care professionals, and reduces the reproductive autonomy of pregnant people.

Given some of these inherent difficulties in characterising severity, and the politics of whose voice should be privileged in that process (even within single genetic conditions), Dive et al. [7]’s call for a shift in focus- from appraisal of the severity of genetic conditions to the utility of information gleaned from genetic tests, brings a new perspective to these debates. Using reproductive carrier screening as an example, Dive et al. [7] suggest that a departure from value judgements about the lives of disabled people, to a consideration of the usefulness of the information derived from genetic screening could reduce some of the tensions around technology use decisions. Whilst perceptions of severity inevitability feed into utility, Dive et al. argue that utility also incorporates factors outwith the condition, for example, the value of the information in the context of a screening programme and the broader healthcare system.

Despite this shift in focus away from the classification of conditions, Knoppers et al.’s [24], paper in this issue however, draws us back to the sheer complexity of genomic results (particularly when delivered in the context of a public health intervention such as newborn screening), and the feasibility of truly informed consent. The potential for ever-widening boundaries of acceptable results (for example, the return of carrier status or late onset untreatable conditions or those of uncertain significance), challenge widely-accepted criteria for inclusion on newborn screening panels. By framing these consent challenges around the novel right of the ‘asymptomatic at-risk newborn to be found’, or perhaps only the *‘pre-symptomatic* child’ to be found [25], the argument for restricting genomic screening to ‘a well curated panel’ (26: 3), with only the most clinically severe conditions [13] with appropriate consent becomes stronger.

Overall, this special issue of papers collectively illuminate the mutability of severity and seriousness, as distinct, but interlinked concepts. Taxonomic systems that have been developed to classify conditions and determine their inclusion on large panel screens can simplify very complex decisions. However, as is demonstrated in this collection, they can also be reductive and stigmatising, require frequent revision (in the context of emerging therapies, healthcare resources and advancing knowledge) and may ultimately reduce parental autonomy [18, 20]. Conditions with wide spectrums of presentation are also particularly challenging to classify within taxonomic systems, and may result in greatly distilled interpretations of seriousness in order to manage the complexity of information [14].

In a departure from labelling both conditions and variants on the sole premise of seriousness or severity, this collection instead invites us to consider the wide range of factors that frame their interpretation across time, place and context, and the value and utility that this information holds for the individual/s. Key constitutive elements are drawn out that are relevant to their conceptualisation [6, 15, 16], with the significance and meaning of each element highly dependent on the values of decision-maker and the type of decision it is used to inform (personal, policy, clinical).

Most importantly, however, this collection offers a springboard for further work in this area that incorporates research in other areas of healthcare where severity or seriousness is used as a threshold concept to allocate resources. For reproductive genomic medicine, the use of severity as a panacea for acceptable technology use obscures its enigmatic foundations, and the inevitable inconsistencies in its interpretation.
